# Improving forecasting accuracy for stock market data using EMD-HW bagging

**DOI:** 10.1371/journal.pone.0199582

**Published:** 2018-07-17

**Authors:** Ahmad M. Awajan, Mohd Tahir Ismail, S. AL Wadi

**Affiliations:** 1 Department of Mathematics, Al Hussien bin Talal University, Ma’an, Jordan; 2 School of Mathematical Sciences, University Science Malaysia, Penang, Malaysia; 3 Department of Risk Management and Insurance, The University of Jordan, Amman, Jordan; Central South University, CHINA

## Abstract

Many researchers documented that the stock market data are nonstationary and nonlinear time series data. In this study, we use EMD-HW bagging method for nonstationary and nonlinear time series forecasting. The EMD-HW bagging method is based on the empirical mode decomposition (EMD), the moving block bootstrap and the Holt-Winter. The stock market time series of six countries are used to compare EMD-HW bagging method. This comparison is based on five forecasting error measurements. The comparison shows that the forecasting results of EMD-HW bagging are more accurate than the forecasting results of the fourteen selected methods.

## Introduction

In financial time series analysis, one of the primary issues is modeling and forecasting financial time series, specifically stock market index. This is because these data are nonlinear and nonstationary. Moreover, it has a height heteroscedasticity. Bagging forecasting is used in this study to overcome these problems. Bagging forecasting, as presented by [1], is a method for generating multiple versions of forecasting and using these versions of forecasting to obtain an aggregated forecasting. Then the average of these versions of forecasting was evaluated.

A bootstrap aggregation of exponential smoothing (EXP) method was presented by [2]. Box-Cox transformation, STL decomposition, and moving block bootstrap (MBB) were used in this study. The M3 data competition (M3 data competition are 3003 time series. The competition results in [3] study are used in comparative papers.) were used to compare this method with original EXP model. In addition as in [4], the M3 data competition were used to compare their method. In this method, the ETS (ExponenTial Smoothing methods) and autoregressive (AR) model with a sieve bootstrap methods were applied. In [5], the Multi-channel Singular Spectrum Analysis (MSSA) technique with HW-Bootstrap method was used to forecast the Natural Inflow Energy time series in Brazil. [6] proposed a bootstrap aggregation (bagging) methodology to reduce the variance of tourism demand forecasting. [7] used the bagging HW method to obtain highly-accurate air transportation demand forecasts. [8] suggested the bagging of STL, Box.Cox, MBB, and ARMA methods to forecasting electric energy demand. Table 1 presents the summary of this literature review.

**Table 1:** Related work used bootstrap in point forecasting technique.

Cite	Method	Data Category
[2]	STL-Box.Cox-MBB-EXP	M3 competition
[4]	ETS-AR-sieve.BOOT	M3 competition
[5]	MSSA-Boot-HW	Natural Inflow Energy
[6]	Resembling-GETS	Tourism demand
[7]	STL-HW	Air transportation demand
[8]	STL-Box.Cox-MBB-ARMA	Electric energy demand

With regard to all those literature reviews, we use the construction of EMD-HW bagging to forecast the stock market data. In order to assess the performance of forecasting, the EMD-HW bagging is compared with fourteen different forecasting models. The experimental results show that the EMD-HW bagging is superior to other methods in terms of RMSE, MAE, MAPE, TheilU, and MASE.

Therefore, the significant of this research article can be summarized. For instance, after an intensive research in the financial forecasting literature, a lot of research papers that have been conducted in forecasting the content of stock market data such as [9], [10], and [11]. Also, most of the articles have used the mentioned models directly without any combination such as [12] and [13].

Section 1 introduces methods that are used in this paper which are EMD, IMF, HW, fourier transform, and MBB. Section 2 presented the proposed methodology and the data that was used. Section 3 analyzes the data with a discussion on the result showing the capability of EMD-HW bagging. Finally, in last Section, some concluding remarks were addressed.

## 1 Methodology

In this section, the various steps for the implementation of the EMD-HW bagging method will be described in details. These methods are Empirical Mode Decomposition (EMD), IMF, HW, Fourier Transform (FT), and Moving Block bootstrap (MBB).

### 1.1 Empirical mode decomposition (EMD)

EMD was described in 1998 by [14], and this method has been applied in [15], [16], [17], [18], [19], [20], [21], and [22]. The main idea of EMD is the decomposing of nonlinear and non-stationary time series data into several of simple time series data. It analyzes time series by keeping the time domain of the signal. It supplies a strong and adaptive process to decompose a time series into a combination of time series that is known as Intrinsic Mode Functions (IMF) and residual. Later, the original signal can be constructed back as the follows:

$$\begin{array}{c} x\left(t\right)=\sum_{i=1}^{n}IMF_{i}\left(t\right)+r\left(t\right) \end{array}$$ (1)

where *x*(*t*) represents the original time series, *r*(*t*) represents the residue of the original time series data decomposition, and *IMF*_*i*_ represent the *i*^*th*^ intrinsic mode function (IMF) series.

However, the EMD technology has a number of limitations in its algorithm. First, the theoretical base is not fully established, and most of the steps of the EMD methodology ignore mathematical expressions. The second limitation is related to the sensitivity toward endpoint treatments (i.e., boundary effect) when using an EMD algorithm [23], and many studies were developed using the EMD methodology to overcome this limitation. Such as, [24] accompanied local polynomial quantile regression with a sifting process for automatic boundary correction. The third limitation is related to mode mixing [25]. The methodology of EMD-HW bagging method able overcomes these limitations with the basic EMD. In the other word, there are a number of studies used extension method (developed method) for the EMD in forecasting method such as [26] and [27], but EMD-HW bagging does not need to use any of these developed methods.

In order to estimate these IMFs, the following steps should be initiated and the process is called the sifting process of time series x(t) [14]. Thus, this is shown below:

1. Taking the original time series *x*(*t*) by assuming that the iteration index value is *i* = 1.
2. Then, evaluating all the local extrema values of the time series *x*(*t*).
3. After that, form the local upper envelope function *u*(*t*) by connecting all local upper values using a cubic spline line. In a similar way, form the local lower envelope *l*(*t*), and then form the mean function *m*(*t*) by using the following;
  $$\begin{array}{c} \;m\left(t\right)=\frac{u(t)+l(t)}{2}\; \end{array}$$ (2)
4. Next, define a new function *h*(*t*) using *m*(*t*) and *x*(*t*) on this formula:
  $$\begin{array}{c} h(t)=x(t)-m(t) \end{array}$$ (3)
  Check the function *h*(*t*) which is an *IMF*, according to *IMF* conditions:
  - The difference between the number of local extreme points and the number of crows-zero points is less than or equal to 1.
  - The absolute value of the mean function is less than *ε*, where *ε* is a very small positive number which is close to zero. Sometime, it is equal to zero.
    (shown in the second part of this section). If the function *h*(*t*) has satisfied *IMF* conditions, then go to step 5. If not, go back to step 2 and renew the value of *x*(*t*) such that it becomes *h*(*t*).
5. Save the result of the *IMF* obtained from the last step, and renew *i* value such that it becomes *i* = *i* + 1, and it attains the residue function *r*(*t*) using the *IMF* and *x*(*t*) by the formula.
  $$\begin{array}{c} \;IMF_{i}\left(t\right)=h\left(t\right)\Rightarrow r_{i+1}\left(t\right)=x\left(t\right)-IMF_{i}\left(t\right) \end{array}$$ (4)
6. Finally, if *r*(*t*) is a monotonic or constant function, then save *r*(*t*) as residue and all the *IMF*s obtained. If *r*(*t*) is not monotonic or constant function, return to step 2.

Real time series data application of most prediction methods needs high degrees of programming for implement of bootstrap [28]. The steps 1 to 6 which were discussed above allow the sifting process to separate time-altering signal features.

### 1.2 Holt-Winter (HW)

More than fifty five years ago, the basic formula of the Holt-Winter (HW) model or Triple Exponential Smoothing have been presented by [29] and [30]. The HW forecasting procedure is a variant of exponential smoothing. HW is simple Which does not need high data-storage requirements and is easily automated. Moreover, HW is particularly suitable for producing short-term forecasts for sales or demand time-series data. In this method, the recent observations have effect which is more robust than old observations in forecasting value.

The HW was employed to short-term forecasting in many studies in literature. Such as; [31] used the HW method in short-term forecasting Ionospheric delay. The HW method used in short-term load forecasting by [32], HW was performing better than six selected forecasting methods. [33] used HW model to forecast electricity demand and concluded that the HW outperform as compared to well-fitted ARIMA models. [34] forecasted the short-term electricity demand for UK and concluded that the HW gives good forecasting results.

Two Holt-Winter models which were described in this study are the Multiplicative Model and the Additive Model. However, the seasonal component determines whether the additive or multiplicative model will be used [35]. Mathematically, the additive Holt-Winters forecasting function is defined by the following:

$$\begin{array}{c} {\hat{y}}_{t+h/t}=a_{t}+h*b_{t}+s_{t-p+1+(h-1)mod(p)}, \end{array}$$ (5)

where *a*_*t*_, *b*_*t*_ and *s*_*t*_ are given by

$$\begin{array}{c} a_{t}=\alpha \left(y_{t}-s_{t-p}\right)+\left(1-\alpha \right)\left(a_{t-1}+b_{t-1}\right)\\ b_{t}=\beta \left(a_{t}-a_{t-1}\right)+\left(1-\beta \right)b_{t-1}\\ s_{t}=\gamma \left(y_{t}-a_{t}\right)+\left(1-\gamma \right)s_{t-p} \end{array}$$

And the multiplicative Holt-Winters forecasting function is defined by the following:

$$\begin{array}{c} {\hat{y}}_{t+h/t}=\left(a_{t}+h*b_{t}\right)*s_{t-p+1+(h-1)mod(p)}, \end{array}$$ (6)

where *a*_*t*_, *b*_*t*_, and *s*_*t*_ are given by

$$\begin{array}{c} a_{t}=\alpha \left(y_{t}/s_{t-p}\right)+\left(1-\alpha \right)\left(a_{t-1}+b_{t-1}\right)\\ b_{t}=\beta \left(a_{t}-a_{t-1}\right)+\left(1-\beta \right)b_{t-1}\\ s_{t}=\gamma \left(y_{t}/a_{t}\right)+\left(1-\gamma \right)s_{t-p} \end{array}$$

where *a*_*t*_, *b*_*t*_, and *s*_*t*_ represent the level, slope, and seasonal of series at time *t*, respectively. Also, *p* represents the number of seasons in a year. The constants *α*, *β* and *γ* are smoothing parameters in the [0, 1]-interval, *h* is the forecast horizon. This method uses the maximum likelihood function to estimate the starting parameters and then it may estimate iteratively all the parameters in forecasting future values of time series. The data in *x*(*t*) are required to be non-zero for a multiplicative model, but it makes more sense if they are all positive.

### 1.3 Moving block bootstrap (MBB)

The moving block bootstrap (MBB) formulated the first structural form in short by [36]. Thus it was known as a general bootstrap method. Now we describe the MBB suppose that {*X*_*t*_}_*t* ∈ *N*_ is a stationary weakly dependent time series and that {*X*_1_, …, *X*_*n*_} ≡ **X**_*n*_ are observed. Let *ℓ* be an integer satisfying 1 < *ℓ* < *n* This gives;

$$\begin{array}{c} \ell /n\to 0\;\;\;as\;\;\;n,\ell \to \infty . \end{array}$$ (7)

Here, the overlapping blocks **B**_1_, …, **B**_*N*_ of length *ℓ* is contained in **X**_*n*_ as:

$$\begin{array}{llllllll} B_{1}= & (X_{1}, & X_{2}, & \ldots , & X_{\ell }) & & & \\ B_{2}= & & (X_{2}, & X_{3}, & \ldots , & X_{\ell +1}) & \\ & & & \ldots & & & \\ B_{N}= & & & (X_{N}, & \ldots , & X_{n-1}, & X_{n}), \end{array}$$

where *N* = *n* − *ℓ* + 1. Let *k* = [*n*/*ℓ*], where for any real number x, [*x*] denotes the least integer greater than or equal to x. Thus, *n* = *ℓ* * *k*. To generate the MBB samples, we selected *k* blocks at random with replacement from the collection {**B**_1_, **B**_2_, …, **B**_*N*_}. Since each resampled block has *ℓ* elements, concatenating the elements of the *k* resampled blocks serially yields *ℓ* * *k* bootstrap observations $X_{1}^{*},\ldots ,X_{n}^{*}$.

### 1.4 Fourier transform

The Fourier transform is the converting of the time series from time domain to frequency domain. In this study, a fast Fourier transform (FFT) was used. The fast Fourier transform is a computational procedure for calculating the finite discrete Fourier transform of a time series. Mathematically, the discrete Fourier transform of finite length is defined as presented by [37]. Let *x*_0_, …., *x*_*N*−1_ be a time series with N observation.

$$\begin{array}{c} X_{k}\;=\sum_{n=0}^{N-1}x_{n}e^{-i2\pi k\frac{n}{N}};\;k=0,\ldots ,N-1. \end{array}$$ (8)

### 1.5 Quantile regression (QR)

The quantile regression was presented [38]. Theoretically, let *Y* be a real valued random variable with cumulative distribution function *F*_*Y*_(*y*) = *P*(*Y* ≤ *y*). The *τ*^*th*^ quantile of Y is given by:

$$\begin{array}{c} Q_{Y}\left(\tau \right)=F_{Y}^{-1}\left(\tau \right)=\inf\{y:F_{Y}\left(y\right)\geq \tau \}. \end{array}$$ (9)

where *τ* ∈ [0, 1].

### 1.6 Statistical techniques for consideration methods

In this study. The EMD-HW bagging method was compared with fourteen methods. Traditional HW method, a hybrid EMD-HW without bagging, ARIMA models, Structural Time Series, Theta method, Exponential smoothing state space method (ETS), and Random Walk method (RW) are used to validate the forecasting performance of EMD-HW bagging. These statistical methods were selected based on their performance in forecasting competitions and other empirical applications, as well as based on their ability to capture salient features of the data.

ARIMA (Autoregressive Integrated Moving Average) or Box-Jenkins models are generally denoted ARIMA(*p*, *d*, *q*). Where parameters *p*, *d*, and *q* are non-negative integers, *p* is the order of the Autoregressive model, *d* is the degree of differencing, and *q* is the order of the Moving-average model. Recently, ARIMA has been employed by several studies in forecasting financial time series such as [39]. He applied ARIMA to forecast the cultivated area and production of maize in Nigeria.

ETS was applied to get short-term solar irradiance forecasting in [40]. The results show that the ETS method majorally has better performance than naive forecasting methods. [41] presented that the forecasts obtained using the Theta method are equivalent to simple exponential smoothing with drift. Also it shows that this method is the best performing method in the M3-Competition [3]. Structural Time Series is applied by [42] on Inbound tourism to New Zealand from selected countries. This method has outperformed the naive process. A random walk (RW) is a process where the current value of a variable is composed of the past value with adding an error term defined as a white noise. It was first studied several hundred years ago as models for games of chance. Recently, [43] have shown that the random walk model turns out to be a hard to beat benchmark in forecasting the CEE exchange rates. While in [44], a variable drift term with the random walk process was applied. This was estimated using a Kalman filter. A Support Vector Machine (SVM) is a discriminative classifier formally defined by a separating hyperplane, the foundations of SVM have been developed by [45]. The Neural Networks have been designed to imitate the biological neural networks that constitute the human brain. In forecasting time series, the historical incidence is sent into the input neurons, and corresponding forecasting incidence is generated from the output neurons after the network is adequately trained [46]. This simple statistical process was shown to perform better than all the three models that were selected in out-of-sample forecasts.

## 2 Data and methodology

This section contains two parts. The first part is about the data that are used to implement the proposed methodology, while the second part presents the proposed methodology EMD-HW bagging with detailed description.

### 2.1 Data

In this study, the daily stock market data of six countries were used. These countries are Australia, France, Malaysia, Netherlands, Sri Lanka and US-S&P 500. The selection of these data was based on the fact that these data are nonlinear and nonstationary time series with a high heteroscedasticity. Table 2 presents the names of countries with the basic statistics for each country, where SD is Standard Deviation, Sk is Skewness, Kts is Kurtosis, N is Number of observations, and Nimf is the number of IMFs.

**Table 2:** Data descriptive statistics.

Country	Mean	Median	Min	Max	SD	SK	Kts	N. IMF	N	KPSS p.value	RESET p.value	BP p.value
**Australia**	4928.42	4939.35	3927.6	5954.8	483.66	0.03	-1.05	7	1498	<.01	<.01	<.01
**France**	3968.26	3939.82	2781.68	5268.91	557.54	0.21	-0.6	7	1516	<.01	<.01	<.01
**Malaysia**	1638.2	1643.89	1072.69	1892.65	164.52	-0.4	-0.68	7	1459	<.01	<.01	<.01
**Netherlands**	370.77	355.92	263.44	509.24	56.19	0.65	-0.32	6	1516	<.01	<.01	<.01
**Sri Lanka**	6208.45	6238.71	3691.04	7811.82	932.63	-0.66	-0.13	7	1431	<.01	<.01	<.01
**USSP500**	1579.25	1493.69	1022.58	2130.82	344.31	0.2	-1.44	6	1490	<.01	<.01	<.01

The KPSS (Kwiatkowski-Phillips-Schmidt-Shin by [47]), RESET (Ramsey Regression Equation Specification Error Test by [48]), and BP (Breusch-Pagan test by [49]), were used to test the nonstationary, nonlinear and heteroscedasticity, respectively. According to p-value for all these tests (< .05), all these stock market are significantly nonlinear and nonstationary with high heteroscedasticity. The data were extracted from the Yahoo finance website.

**Fig 1:**
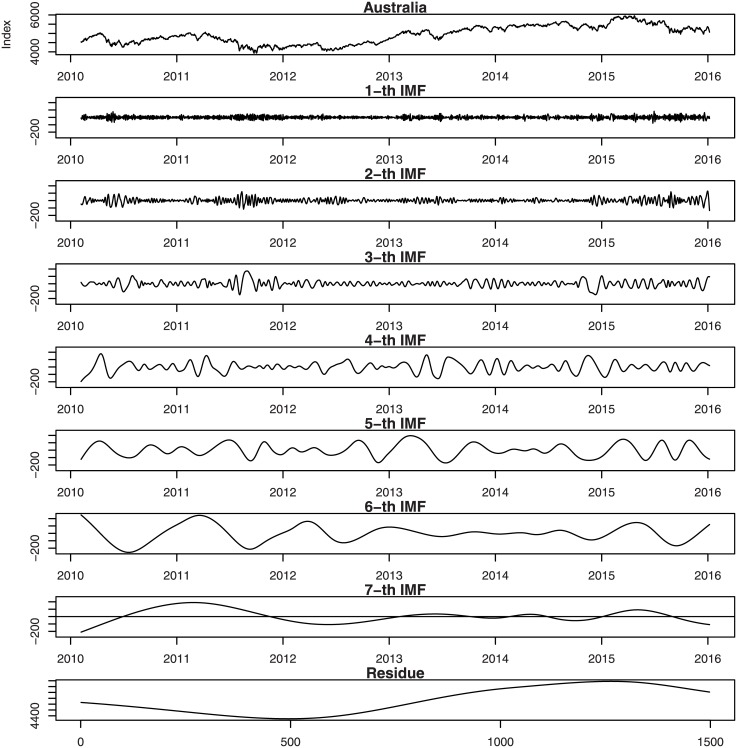
Australia stock market with its IMFs and residue plots.

**Fig 2:**
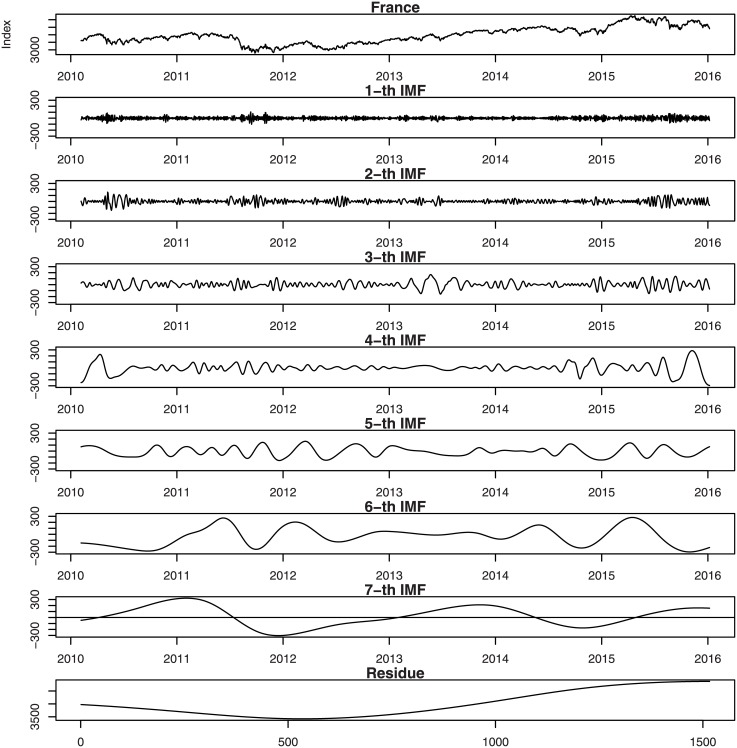
France stock market with its IMFs and residue plots.

**Fig 3:**
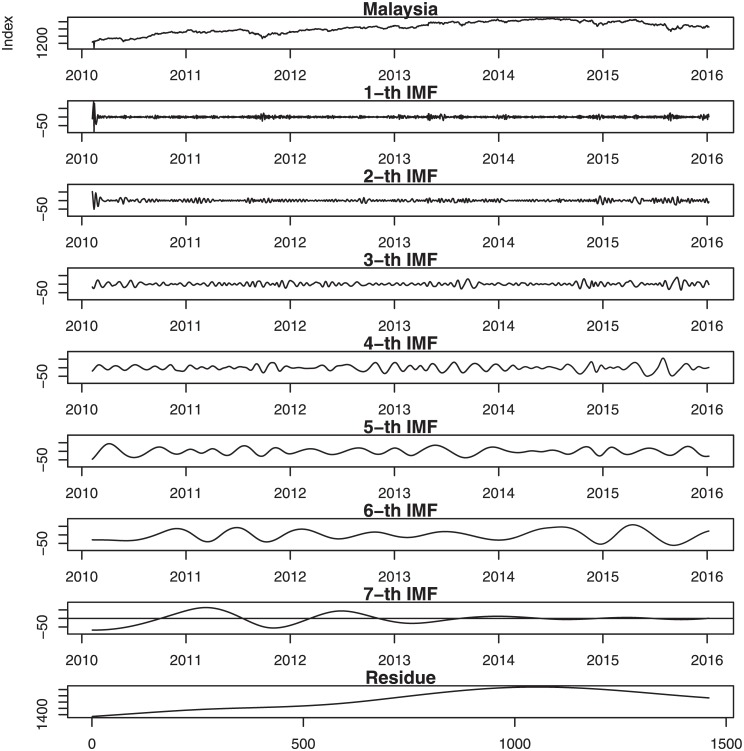
Malaysia stock market with its IMFs and residue plots.

**Fig 4:**
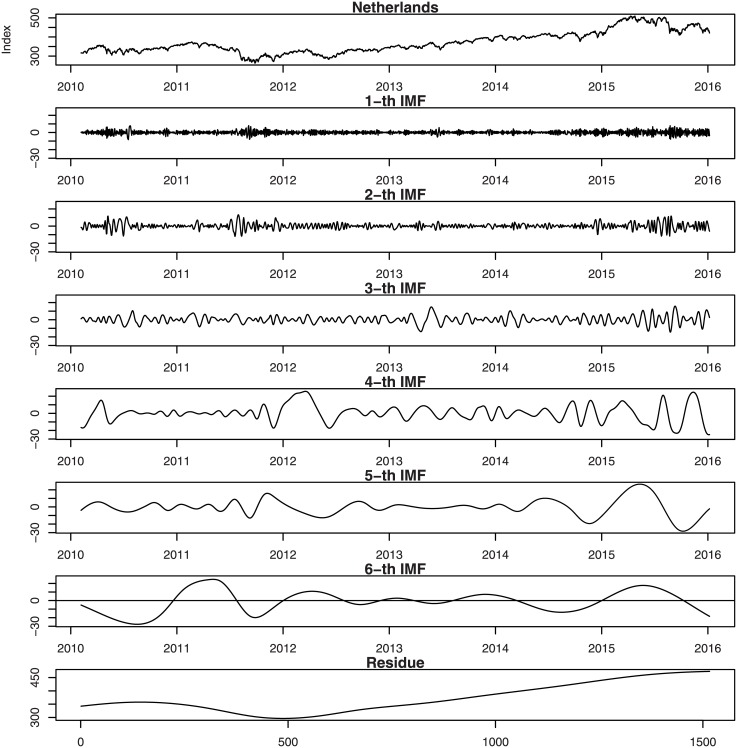
Netherlands stock market with its IMFs and residue plots.

**Fig 5:**
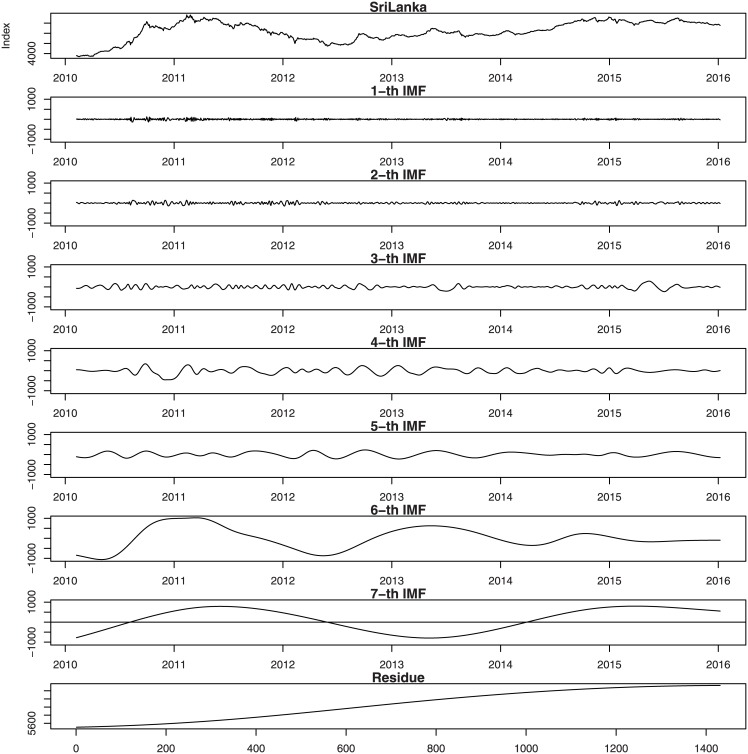
Sri Lanka stock market with its IMFs and residue plots.

**Fig 6:**
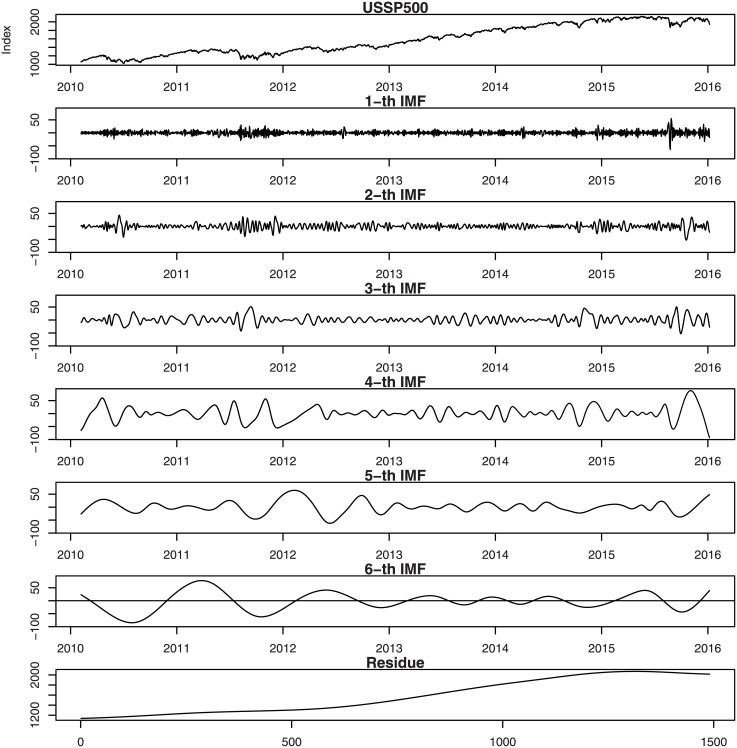
US-SP500 stock market with its IMFs and residue plots.

Figs 1, 2, 3, 4, 5, and 6 show the stock market with its IMFs and residue plots of these countries, respectively. The daily closing prices are used as a general measure of the stock market over the past six years. The whole data set -for each country- covers the period from 9 February 2010 to 7 January 2016, a long sample period renders the results of our test more convincing. Dickey-Fuller test was introduced [47] and was implemented on time series data to prove non-stationarity.

The data set are divided into two parts. The first part (n observations) is used to determine the specifications of the models and parameters. The second part, on the other hand (h observations), is reserved for out-of-sample evaluation. This part is used for the comparison of performances among various forecasting models. The Malaysian stock market data (KLSM) are taken as an example. Here, the number observation is N = 1459. The first part n = 1458, 1457, 1456, 1455, 1454, and 1453 and the second part h = 1, 2, 3, 4, 5, and 6 respectively, are used.

In the other words, we take six different periods of forecast steps. These periods are one day, two days, three days, four days, five days, and six days. This means we use six different training periods for each data.

### 2.2 Methodology

The EMD-HW bagging methodology [50] consists of six steps.

1. The QR—with *τ* = 0.5—is applied on the original time series *x*_*t*_ to decompose *x*_*t*_ into noisy series *E*(*t*) and regression line *RL*. The use of EMD on noisy series *E*(*t*). On this step, the IMFs and residue are obtained. This step is called EMD-QR.
2. The Fourier transform are applied on IMFs from time domain to transform to frequency domain.
3. According the transformation results from the last step, the *IMF*s are clustered into two clusters high and low frequency (*HF* and *LF*) with 0.02 threshold criteria.
4. The high frequency components are aggregate to gather to get a high frequency time series. This time series is bootstrapped using MBB with length *ℓ* observations (The optimal block length is estimated automatically for each time series by [51].). Also, the value of *B* (In theory, the ideal bootstrap will be obtained when the *B* value goes to infinity [52]. There is no a formal method for selecting the *B* value in the literature [53]. So, selecting *B* value is still a problem so that the time spent for bootstrapping depends on *B* value as well as on the sample length [54].) is selected in this step. The value of *B* is fixed in this study at *B* = 2000 provides a reasonable approximation see [55]. Which they are 1999 new series with the original series.
5. The new component was added to low frequency IMFs, linear regression and residue again to generating *B* synthetic series.
6. The EMD-QR is applied on each of the bootstrapped time series. Then, *h* point ahead is forecasted using the *HW* model for regression line and residual and all low frequency IMF’s. Also, it adds all the forecasting result together.
7. In the last step, *B* forecasting point were calculated. The resulting forecasts were combined using the median (The median is the aggregating measure adopted as it is less sensitive to outliers [7].) to get the point forecasting for the original time series *x*(*t*).
8. The forecasting result was compared with fourteen forecasting technique based on five error measurements.

**Fig 7:**
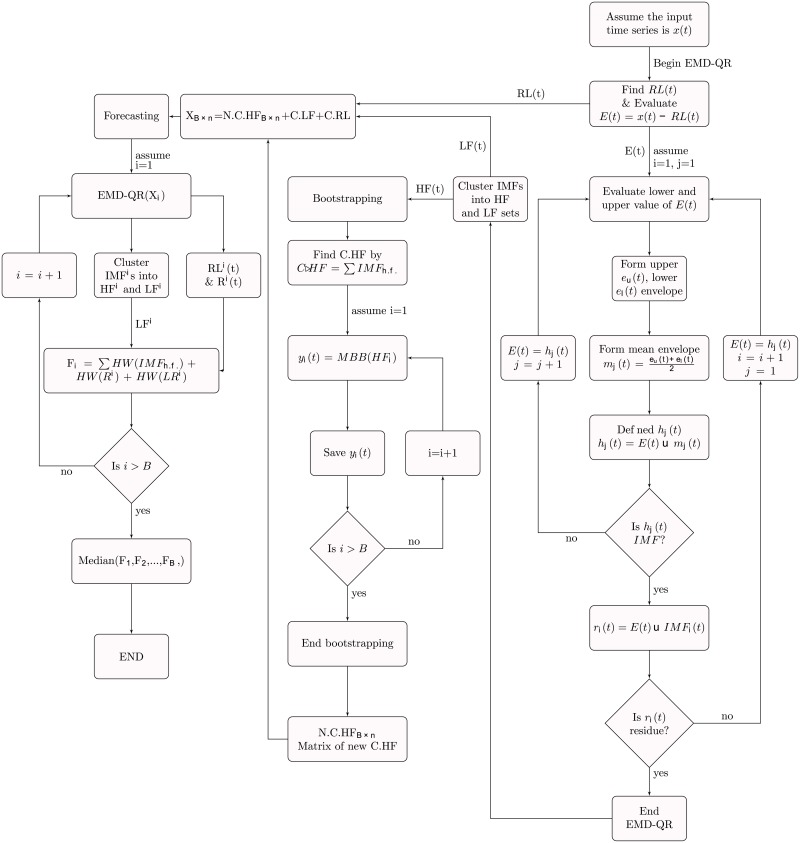
Flow chart of EMD-HW bagging.

As mentioned before, for each data there are six different of forecast periods. For all these periods the same steps are used. In the other word, the different forecast steps do not affect the forecasting model selection or parameter selection. Fig 7 present the flow chart of EMD-HW bagging.

## 3 Results and discussion

In this study, we present the bagging for EMD and HW technique (EMD-HW bagging) using HW, EMD, and MBB. The stock market data of six countries are used to evaluate the forecasting accuracy of the EMD-HW bagging method. Fourteen forecasting models are used in order to validate the forecasting performance EMD-HW bagging method. Root Mean Squared Error (RMSE), Mean Absolute Error (MAE), Mean Absolute Percentage Error (MAPE), Theil’s U-statistic (TheilU), and Mean Absolute Scaled Error(MASE) will be utilized to evaluate the forecasting accuracy for each method. Eqs (10), (11), (12), (13), and (14) show the formula of RMSE, MAE, MAPE, MASE, and TheilU, respectively. Where $\hat{y_{i}}$ is the forecast value of the variable *y* at time period *i* based on the knowledge of the actual series values.

$$\begin{array}{c} RMSE=\sqrt{\frac{1}{n}\sum_{i=1}^{n}{\left(y_{i}-{\hat{y}}_{i}\right)}^{2}} \end{array}$$ (10)

$$\begin{array}{c} MAE=\frac{1}{n}\sum_{i=1}^{n}|y_{i}-{\hat{y}}_{i}| \end{array}$$ (11)

$$\begin{array}{c} MAPE=\frac{1}{n}\sum_{i=1}^{n}\frac{\left|y_{i}-{\hat{y}}_{i}\right|}{y_{i}}.100\% \end{array}$$ (12)

$$\begin{array}{c} MASE=\frac{1}{n}\sum_{i=1}^{n}\left(\frac{\left|y_{i}-{\hat{y}}_{i}\right|}{\frac{1}{n-1}\sum_{i=2}^{n}\left|y_{i}-y_{i-1}\right|}\right) \end{array}$$ (13)

$$\begin{array}{c} TheilU=\frac{\sqrt{\sum_{i=1}^{n-1}{\left(\frac{{\hat{y}}_{i+1}-y_{i+1}}{y_{i}}\right)}^{2}}}{\sqrt{\sum_{i=1}^{n-1}{\left(\frac{y_{i+1}-y_{i}}{y_{i}}\right)}^{2}}} \end{array}$$ (14)

Tables 3 and 4 present the average of RMSE, MAE, MAPE, TheilU, and MASE for the forecasting of EMD-HW bagging and fourteen method at h = 1, 2, 3, 4, 5, and 6 for the stock market data of six countries. These are Australia, France, Malaysia, Netherlands, Sri Lanka, and US-S&P 500.

**Table 3:** The average of five error measures for EMD-HW bagging and fourteen forecasting methods at 1 to 6 for Australia, France, and Malaysia stock market.

Country	Method	RMSE	MAE	MAPE	TheilU	MASE
Australia	HW	144.618	125.059	2.439	2.104	1.619
	EXP	125.536	104.889	2.049	1.831	1.343
	Meanf	146.774	126.863	2.475	2.135	1.641
	ARIMA	144.260	124.882	2.436	2.107	1.626
	Thita	146.529	126.663	2.471	2.130	1.638
	RW	145.573	125.870	2.455	2.115	1.626
	B.EXP.AR	167.393	153.376	2.991	2.057	1.713
	B.EXP.STR	135.104	114.712	2.244	1.672	1.203
	B.HW	123.225	107.078	2.089	1.601	1.243
	NNETAR	163.346	141.329	2.654	2.411	1.866
	SVM	369.596	361.843	7.521	4.297	5.039
	EMD.NNETAR	137.164	117.948	2.238	1.749	1.310
	EMD.SVM	254.104	242.929	4.926	2.882	3.462
	EMD.ARIMA	167.549	149.114	2.802	2.210	1.732
	EMD-HW bagging	92.707	81.391	1.565	1.064	1.128
France	HW	131.292	118.930	2.667	2.389	2.077
	EXP	218.606	208.373	4.672	3.622	3.327
	Meanf	130.711	118.399	2.655	2.377	2.066
	ARIMA	130.491	118.203	2.651	2.370	2.053
	Thita	130.223	117.953	2.645	2.368	2.058
	RW	129.010	116.901	2.622	2.345	2.040
	B.EXP.AR	168.347	158.299	3.554	2.848	2.562
	B.EXP.STR	414.818	409.442	9.176	6.274	6.252
	B.HW	390.128	385.050	8.625	5.938	5.933
	NNETAR	183.216	167.317	3.572	3.362	2.936
	svm	479.517	475.970	11.893	6.803	7.795
	EMD.NNETAR	124.844	113.978	2.477	1.900	1.608
	EMD.svm	329.900	324.963	7.827	4.636	5.434
	EMD.ARIMA	440.666	437.597	8.874	7.002	7.213
	EMD-HW bagging	119.635	107.026	2.405	2.122	1.781
Malaysia	HW	19.374	17.894	1.078	1.534	1.656
	EXP	15.813	13.652	0.818	1.174	1.393
	Meanf	18.944	17.565	1.058	1.489	1.619
	ARIMA	18.558	17.010	1.025	1.479	1.596
	Thita	18.691	17.324	1.044	1.468	1.598
	RW	19.205	17.671	1.065	1.524	1.650
	B.EXP.AR	19.162	17.945	1.080	1.721	1.924
	B.EXP.STR	23.243	22.457	1.347	1.655	2.100
	B.HW	17.032	14.717	0.881	1.059	1.362
	NNETAR	21.063	19.632	1.163	1.657	1.800
	SVM	63.872	63.202	3.951	4.762	5.900
	EMD.NNETAR	15.387	13.821	0.826	1.062	1.152
	EMD.SVM	62.626	61.886	3.865	4.737	5.819
	EMD.ARIMA	21.678	19.96	1.186	1.668	1.805
	EMD-HW bagging	18.991	16.982	1.017	1.337	1.586

**Table 4:** The average of five error measures for EMD-HW bagging and fourteen forecasting methods at 1 to 6 for Netherlands, SriLanka, and US-S&P 500 stock market.

Country	Method	RMSE	MAE	MAPE	TheilU	MASE
Netherlands	HW	12.126	10.977	2.577	2.101	1.728
	EXP	22.573	21.564	5.058	3.585	3.160
	Meanf	12.158	11.006	2.583	2.107	1.732
	ARIMA	11.946	10.831	2.542	2.075	1.712
	Thita	12.087	10.941	2.568	2.093	1.720
	RW	11.964	10.829	2.542	2.070	1.702
	B.EXP.AR	14.642	13.589	3.193	2.356	1.978
	B.EXP.STR	44.289	43.847	10.273	6.476	6.271
	B.HW	39.910	39.470	9.244	5.923	5.717
	NNETAR	17.047	15.379	3.438	3.035	2.480
	SVM	108.292	108.146	33.811	15.247	16.245
	EMD.NNETAR	13.347	11.962	2.719	2.206	1.723
	EMD.SVM	119.880	119.735	39.080	16.624	17.639
	EMD.ARIMA	41.359	40.587	8.578	6.394	6.043
	EMD-HW bagging	11.437	10.141	2.384	1.894	1.492
SriLanka	HW	47.952	42.683	0.627	1.705	1.324
	EXP	121.815	120.398	1.768	3.783	3.617
	Meanf	48.716	43.320	0.637	1.741	1.350
	ARIMA	53.559	47.088	0.692	1.971	1.503
	Thita	55.244	48.847	0.718	1.985	1.518
	RW	54.065	47.779	0.703	1.938	1.482
	B.EXP.AR	58.807	51.096	0.7507	2.0317	1.563
	B.EXP.STR	170.433	158.357	2.326	4.985	4.28
	B.HW	131.494	127.006	1.868	3.605	3.206
	NNETAR	80.303	70.981	1.030	2.933	2.249
	SVM	1,451.550	1,451.226	27.051	43.157	44.735
	EMD.NNETAR	34.024	31.385	0.460	0.995	0.908
	EMD.SVM	782.748	782.155	12.963	23.304	24.338
	EMD.ARIMA	285.002	284.133	4.351	8.489	9.002
	EMD-HW bagging	48.222	40.688	0.599	1.665	1.187
US-S&P 500	HW	58.927	51.949	2.638	2.105	1.788
	EXP	59.068	52.113	2.645	2.112	1.799
	Meanf	60.645	53.453	2.714	2.159	1.831
	ARIMA	59.644	52.442	2.664	2.103	1.770
	Thita	59.958	52.839	2.683	2.133	1.807
	RW	58.858	51.865	2.634	2.093	1.772
	B.EXP.AR	73.690	67.722	3.441	2.394	2.110
	B.EXP.STR	103.250	97.943	4.968	3.253	3.045
	B.HW	97.697	92.714	4.709	2.950	2.729
	NNETAR	45.434	41.517	2.020	2.399	2.166
	SVM	344.596	344.075	20.626	15.321	17.768
	EMD.NNETAR	88.802	83.261	3.958	4.692	4.441
	EMD.SVM	392.473	392.006	24.190	17.386	20.099
	EMD.ARIMA	60.651	53.596	2.632	2.044	1.709
	EMD-HW bagging	62.089	55.421	2.823	1.907	1.567

This indicates that the forecast accuracy for EMD-HW bagging method is better than the fourteen forecasting models. As a conclusion, this proved that the EMD-HW bagging method is giving a better forecasting result than selected models. The authors believe that our conclusions of robust performances of improving the forecasting accuracy for EMD-HW bagging will be useful to the governments’ decision makers and the investments. The authors plan to used EMD-HW bagging to forecast other kinds of financial time series such as exchange rate.

## Conclusion

In this study, the EMD-HW bagging is used to forecast daily stock market data of six counties. These countries are Australia, France, Malaysia, Netherlands, Sri Lanka, and US-S&P 500. Consequently, this method is used to forecast nonstationary and nonlinear time series. The result of EMD-HW bagging forecasting technique were compared with forecasting result of six traditional method with three bootstrapping methods. This comparison is based on five forecasting error measurements (RMSE, MAE, MAPE, TheilU, and MASE). In general, the comparison shows that the forecasting results of EMD-HW bagging have more accuracy than the results of these fourteen forecasting methods.

## Supporting information

S1 Fig Displays the actual data for Australia stock market from 2010.02.09 to 2016.01.07 with the its IMFs and residues. (CSV)

S2 Fig Displays the actual data for France stock market from 2010.02.09 to 2016.01.07 with the its IMFs and residues. (CSV)

S3 Fig Displays the actual data for Malaysia stock market from 2010.02.09 to 2016.01.07 with the its IMFs and residues. (CSV)

S4 Fig Displays the actual data for Netherlands stock market from 2010.02.09 to 2016.01.07 with the its IMFs and residues. (CSV)

S5 Fig Displays the actual data for Sri Lanka stock market from 2010.02.09 to 2016.01.07 with the its IMFs and residues. (CSV)

S6 Fig Displays the actual data for USSP500 stock market from 2010.02.09 to 2016.01.07 with the its IMFs and residues. (CSV)
